# Training PBertKla on an Integrated Multi-Source Dataset with a Machine-Learning Layer for Lysine Lactylation Site Prediction

**DOI:** 10.3390/ijms27135761

**Published:** 2026-06-26

**Authors:** Seung Beom Jin, Junghee Park, Summer Dabin Lee, Ji Hye Han, Seung-Hyun Myung, Kichul Park, Jisoo Yun

**Affiliations:** 1 LNPsolution, 199-9 Dugaebisan-ro, Hongcheon-gun 25114, Gangwon-do, Republic of Korea; sb_jin96@lnpsolution.com (S.B.J.); summer@lnpsolution.com (S.D.L.); 2 Department of Biochemistry and Molecular Biology, Chosun University School of Medicine, 309 Pilmoon-daero, Dong-gu, Gwangju 61452, Republic of Korea; jungheepark@chosun.ac.kr (J.P.); ster8823@naver.com (J.H.H.); myoungsh725@naver.com (S.-H.M.); 3 Institute of Well-Aging Medicare & Chosun University LAMP Center, Chosun University, Gwangju 61452, Republic of Korea; 4 Medical Research Institute & Chosun University LAMP Center, Chosun University, Gwangju 61452, Republic of Korea

**Keywords:** lysine lactylation, post-translational modification prediction, curated benchmark dataset, leakage-controlled validation, protein language model, ProteinBERT, machine learning, AlphaFold

## Abstract

Lysine lactylation (Kla) is a recently discovered post-translational modification implicated in energy metabolism, cellular reprogramming, and disease progression. Here, we train the existing ProteinBERT-based predictor PBertKla on an integrated multi-source dataset and augment it with a lightweight machine-learning (ML) layer over sequence-derived features to predict Kla sites; on a common blind test set, the resulting model (PBertKla + ML) reaches an area under the receiver operating characteristic curve (AUROC) of 0.9126 on the integrated set and is statistically indistinguishable from the strongest available tool (Auto-Kla, DeLong *p* = 0.74) while significantly exceeding a recent ProtBert-based method (PCBert-Kla, *p* = 4 × 10^−15^). Two elements support this result. First, to train and benchmark the model, we assembled and released the largest curated Kla dataset to date, Multi (26,034 samples compiled from nine published sources through a 9-step quality-control pipeline), as a community resource. Second, we validated the model under a leakage-controlled protocol: re-training the complete pipeline under protein-level, 40%-identity homology, and leave-one-study-out splits—each verified to have zero train–test overlap—maintained ≈0.90 AUROC, only 0.6–1.5 percentage points (pp) below the random-split value, confirming genuine generalization rather than memorization. Ablation and SHapley Additive exPlanations (SHAP) analyses locate the predictive signal primarily in the ProteinBERT metafeature, with the ML layer adding a modest but real increment (+0.63 pp over PBertKla alone on Multi; no significant gain on the smaller hepatocellular carcinoma (HCC) set). Finally, an exploratory AlphaFold-based structural case study of FAM210A illustrates how predicted Kla sites distribute across ordered and disordered regions, without claiming a quantitative structure–probability relationship. All trained weights and code are publicly available.

## 1. Introduction

Lysine lactylation (Kla) is a post-translational modification (PTM) first identified in histones by Zhang et al. in 2019 [1], in which lactate, a glycolytic metabolite, is covalently attached to the ε-amino group of lysine residues. Subsequent work by Wan et al. [2] revealed that lactylation extends broadly to non-histone proteins, and Yang et al. [3] demonstrated via genetic code expansion that site-specific lactylation reshapes protein function. Kla has been implicated in energy metabolism, cellular reprogramming, inflammation, and cancer progression [4], and histone acetyltransferase p300/CBP has been identified as a lactylation-catalyzing enzyme [1,5]. Beyond these foundational findings, lactylation has been linked to diverse pathophysiological processes, including the macrophage inflammatory-to-reparatory transition [6] and pulmonary fibrosis [7], and its roles in cancer have been reviewed in detail [8,9]. In hepatocellular carcinoma (HCC)—the source of the principal benchmark used here—lactylome studies have associated Kla with metabolic adaptation [10] and disease progression [11].

Several sequence-based Kla prediction tools have been developed, including FSL-Kla [12], DeepKla [13], and Auto-Kla [14]. More recently, PBertKla [15] applied the ProteinBERT protein language model (pLM) to achieve an area under the receiver operating characteristic curve (AUROC) of 0.884 on an independent test set. PCBert-Kla [16] fuses physicochemical properties with ProtBert in an end-to-end fashion; however, this design requires full model retraining when incorporating new feature types. SAPP [17] integrates AlphaFold2 structural features into PTM predictions, demonstrating that structural features can contribute to PTM prediction. We adopt the ProteinBERT backbone of PBertKla [15] rather than sequence-only protein language models such as ESM2 [18]: retaining the same backbone enables a like-for-like comparison with the prior predictor, while ProteinBERT’s joint sequence–function pretraining provides a functional inductive bias well suited to an enzyme-mediated modification. Structure-aware feature-fusion approaches have likewise been explored for Kla prediction [19].

In this study, we make the following contributions. (1) We train the existing ProteinBERT-based predictor PBertKla on an integrated multi-source dataset and augment it with a lightweight machine-learning classifier over sequence-derived features (AAC, DPC, length); on a common blind test set, the resulting PBertKla + ML model matches the strongest available tool (Auto-Kla) and exceeds a recent ProtBert-based method (PCBert-Kla), and we analyze—via ablation and SHapley Additive exPlanations (SHAP—where its predictive signal originates. (2) To train and benchmark this model, we curate the largest Kla dataset to date, Multi (26,034 samples compiled from nine published sources), through a 9-step quality-control pipeline and release it as a community benchmark. (3) We validate the model under a rigorous leakage-controlled protocol—re-training the entire pipeline under protein-level, homology-reduced, and leave-one-study-out splits—confirming that its performance reflects genuine generalization rather than train–test leakage. (4) We report an exploratory AlphaFold-based structural case study of FAM210A, illustrating how predicted Kla sites distribute across a protein’s ordered and disordered regions. We also document and correct a naive-versus-proper metafeature-generation pitfall encountered when building the ML layer.

## 2. Results

### 2.1. Dataset Construction and Expansion

Two benchmark datasets were used in this study (Table 1). HCC is a human hepatocellular carcinoma Kla dataset from the original PBertKla study [15], comprising 4820 positive and 4820 negative samples (9640 in total). Multi is an expanded multispecies dataset assembled from nine published studies, including human HCC [15], gastric cancer [20], human lung [21], HCC proteomics [22], rat [23], *Toxoplasma gondii* [24], *Phialophora verrucosa* [25], *Botrytis cinerea* [26], and rice [27]. After a 9-step quality-control pipeline (Supplementary Table S1)—comprising source collection; UniProt mapping and sequence retrieval; removal of entries without a retrievable sequence, non-lysine sites, and out-of-length sites; integration with the PBertKla baseline set; exact window-sequence deduplication; window extraction (±22 residues centered on K, 45-mer); 1:1 class balancing; and an 80:20 train/test split—the final dataset contains 13,017 positive and 13,017 negative samples (26,034 total). Homology- and protein-level control between train and test is provided separately by the leakage analysis in Section 2.5.

### 2.2. PBertKla Reproduction

We reproduced PBertKla [15] using an identical HCC dataset and ProteinBERT architecture. Contrary to the reported ACC of 80.3% and AUROC of 0.884, our reproduction achieved an ACC of 78.2% and AUROC of 0.875 within 1–3 percentage points (pp), confirming the validity of the architecture. These differences are attributed to the hyperparameter search ranges and random seeds. The per-fold results are provided in Supplementary Table S2 and Figure S1.

### 2.3. PBertKla + ML: Combining the PBertKla Metafeature with Sequence Features

**Figure 1:**
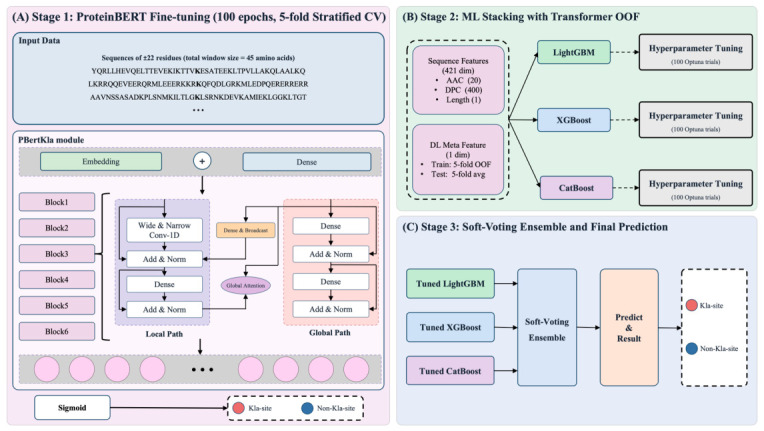
PBertKla + ML three-stage pipeline architecture. (**A**) Stage 1: ProteinBERT 5-fold StratifiedKFold fine-tuning with frozen warmup → full fine-tuning → long-sequence fine-tuning. (**B**) Stage 2: 422-dimensional feature vector (AAC 20 + DPC 400 + length 1 + DL meta 1) input to LightGBM, XGBoost, and CatBoost with Optuna 100-trial optimization. (**C**) Stage 3: Soft-voting ensemble.

The combined PBertKla + ML classifier is built as a three-stage sequential pipeline (Figure 1).

Stage 1 (ProteinBERT Fine-tuning): A pretrained ProteinBERT checkpoint (epoch 92,400/sample 23.5 M, 191 MB) [28] was fine-tuned with 5-fold StratifiedKFold (seed = 42). Each fold proceeded through three phases: (i) frozen warmup—encoder weights frozen, only the classification head trained; (ii) full fine-tuning—all parameters trained with lr = 2 × 10^−3^, batch size 32, sequence length 512, and max 100 epochs (patience = 15, early stopping); and (iii) long-sequence fine-tuning—sequence length extended to 1024 for one additional epoch. OOF predictions for the training set and 5-fold averaged predictions for the test set were saved as deep learning (DL) metafeatures to ensure distribution consistency between the training and test metafeatures (see Section 2.8).

Stage 2 (ML Meta-Classifier): A 422-dimensional feature vector was constructed: amino acid composition (AAC, 20-dim), dipeptide composition (DPC, 400-dim), sequence length (1-dim), and DL metafeature (1-dim, OOF probability from Stage 1). LightGBM, XGBoost, and CatBoost were optimized using Optuna [29], with 100 trials × 5-fold cross-validation (CV) (seed = 42) to maximize the AUC-ROC. The key design principle is modularity: new features, such as ESM2 embeddings and conservation scores, can be appended to the 422-dim vector, and only the ML layer needs to be retrained without modifying the DL backbone. The individual performances and ROC curves of the three ML classifiers are provided in Table S3 and Figure S2.

Stage 3 (Soft-Voting Ensemble): Prediction probabilities from the three ML models were averaged: p_final = (p_LGB + p_XGB + p_CAT)/3, with a threshold of 0.5 for binary classification.

### 2.4. Data Expansion and the Machine-Learning Layer

**Figure 2:**
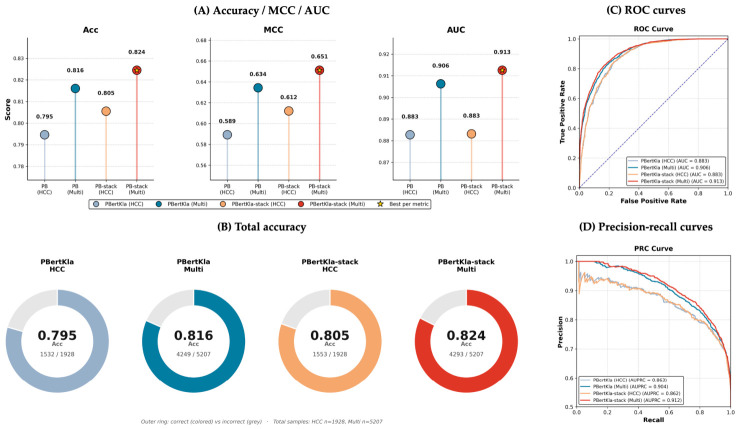
Comprehensive performance comparison of four models. (**A**) Accuracy, MCC, and AUC (★ = best). (**B**) Total accuracy. (**C**) ROC curves. (**D**) Precision-recall curves. PBertKla + ML (Multi) achieved the highest performance among the PBertKla-family configurations evaluated here.

The effects of dataset expansion (HCC → Multi) and DL + ML meta-classifier are summarized in Table 2 and Figure 2. Dataset expansion improved AUROC by +2.36 pp, F1 score by +2.77 pp, and ACC by +2.14 pp (DL-only basis). Adding the ML layer yielded +0.04–0.63 pp in AUROC (noting that the HCC gain of +0.04 pp is within the fold-level variance and not statistically significant). F1 score improvement was +0.80–1.54 pp. Because HCC is a subset of Multi, and the two datasets were split independently with substantial overlap (87.4% of HCC sequences appeared in Multi, and 70.4% of the HCC test set appeared in the Multi train set), the performance difference reflects both expanded training data and differing test set compositions rather than a pure data-scaling effect. Nevertheless, these results indicate that data curation is a more effective strategy than architectural complexity for predicting Kla. The final PBertKla + ML (Multi) achieved AUROC of 0.9126, F1 score of 0.8314, ACC of 0.8245, and MCC of 0.651, the highest among the PBertKla-family configurations evaluated here.

To test whether this overlap inflates performance, we re-evaluated PBertKla and PBertKla + ML on the 571 non-overlapping HCC test windows (clean subset; 186 positive, 385 negative). Both models maintained their performance on these unseen windows (AUROC 0.900 and 0.899) relative to the full test set (0.883 for both), with no degradation. This is consistent with the protein-level leakage-controlled analysis (Section 2.5), which confirms on a larger, stricter split that the reported performance reflects genuine generalization rather than memorization of shared sequences.

To assess statistical robustness, we computed 2000-resample bootstrap 95% confidence intervals (CIs) and DeLong tests for paired AUROC comparison (Figure S4). On HCC, PBertKla + ML reached AUROC 0.8831 (95% CI 0.8677–0.8974) versus PBertKla 0.8827 (0.8676–0.8964); the difference was not significant (ΔAUROC = +0.0004, DeLong *p* = 0.648). On Multi, PBertKla + ML reached 0.9126 (0.9051–0.9196) versus 0.9063 (0.8985–0.9137); the difference was small but significant (ΔAUROC = +0.0063, *p* < 0.0001). These results confirm that the gain from the ML layer, where present, is modest.

### 2.5. Leakage-Controlled Evaluation

**Figure 3:**
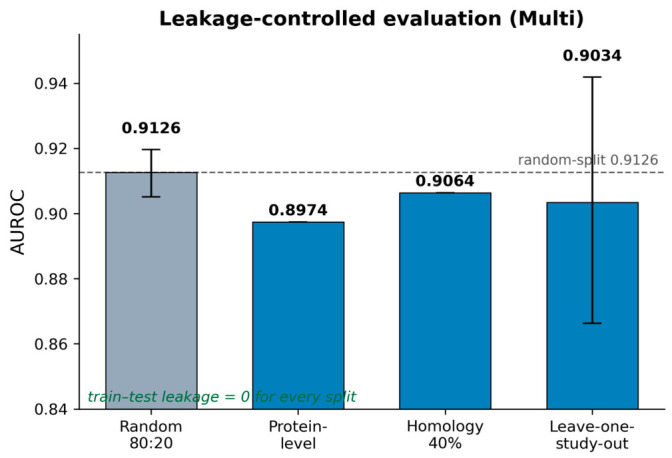
Leakage-controlled evaluation on the Multi benchmark. The full pipeline was re-trained from scratch under three leakage-controlled splits (protein-level, 40%-identity homology, and leave-one-study-out), each with zero train–test overlap, and compared to the random 80:20 split. Ensemble AUROC: random 0.9126, protein-level 0.8974, homology 0.9064, leave-one-study-out 0.9034 (mean of 7 folds; range 0.866–0.942). Error bars: 95% bootstrap CI (random split) and across-fold range (leave-one-study-out); protein- and homology-level points are single leakage-controlled splits. Full values are given in Table S7.

To test whether the reported performance is inflated by train–test leakage, we re-trained the complete pipeline under three leakage-controlled re-splits of the Multi dataset, each removing one leakage path: (i) a protein-level split (all windows from a given UniProt protein assigned to the same side, via GroupShuffleSplit on accession); (ii) a homology-reduced split (all 26,034 windows clustered with CD-HIT at 40% identity and whole clusters assigned to one side, so no train window is >40% identical to any test window); and (iii) a leave-one-study-out evaluation (each of the seven source studies held out in turn). Each split was verified to contain zero shared proteins, clusters, or studies across the train–test boundary (Figure 3; Table S7). Performance was maintained at ≈0.90 AUROC under all three controls (ensemble AUROC 0.8974 protein-level, 0.9064 homology, 0.9034 mean across leave-one-study-out folds), only 0.6–1.5 percentage points below the random-split value (0.9126). This indicates that the reported performance reflects genuine generalization to unseen proteins, homologs, and studies rather than memorization of leaked sequences. As in the random-split analysis, the ML layer did not exceed the best single base model on any split.

### 2.6. Head-to-Head Comparison with Existing Tools

**Figure 4:**
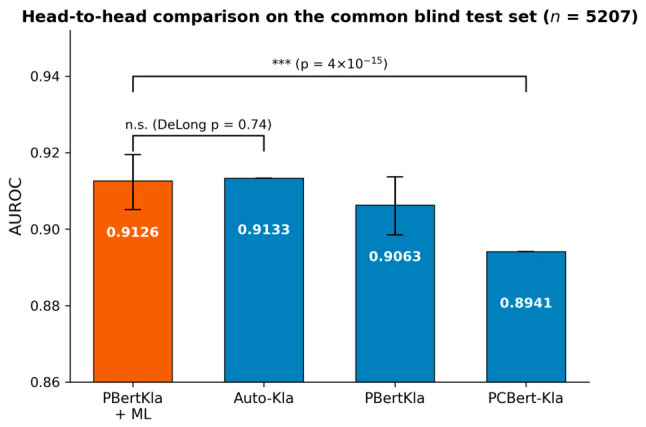
Head-to-head comparison on the common blind test set (*n* = 5207). AUROC of PBertKla + ML (0.9126), Auto-Kla (0.9133), PBertKla (0.9063), and PCBert-Kla (0.8941). PBertKla + ML is statistically indistinguishable from Auto-Kla (DeLong *p* = 0.74) and significantly exceeds PCBert-Kla (DeLong *p* = 4 × 10^−15^). Error bars: 95% bootstrap CI where available. Full values are given in Table S8. *** *p* < 0.001.

To benchmark PBertKla + ML against existing tools on equal footing, we re-trained three competing methods on our Multi training split and evaluated them on the identical blind test set (*n* = 5207), reporting the same metrics (Figure 4; Table S8). Auto-Kla (the current AutoGluon release of the same AutoML approach, as the original 0.5.2/CUDA 11.3 environment is incompatible with current GPUs) reached AUROC 0.9133, statistically indistinguishable from PBertKla + ML (ΔAUROC = +0.0007, DeLong *p* = 0.74). PCBert-Kla (its released ProtBert-4-layer + physicochemical architecture) reached AUROC 0.8941, significantly below PBertKla + ML (ΔAUROC = −0.0185, DeLong *p* = 4 × 10^−15^). DeepKla could not be run faithfully—it is frozen at Python 2.7/Keras 2.0.6 (both end-of-life) with hand-coded custom-attention layers that do not run on current Python/TensorFlow or GPUs, and porting them would alter the published model—so we cite its reported performance (different test set) rather than risk an unfair re-implementation. In summary, PBertKla + ML is statistically indistinguishable from the strongest available tool (Auto-Kla) and significantly exceeds both a recent ProtBert method (PCBert-Kla) and the prior predictor (PBertKla) on a common blind test set; consistent with the ablation analysis, this places its contribution in the curated dataset and modular design rather than a raw accuracy increase.

### 2.7. Ablation: DL Metafeature Contribution

**Figure 5:**
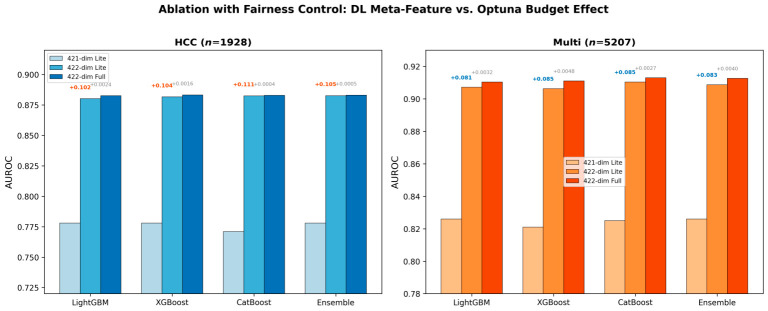
Ablation with fairness control. (**Left**): HCC, (**Right**): Multi. Three conditions: 421-dim Lite (light), 422-dim Lite (medium), 422-dim Full (dark). Orange annotations = DL meta-feature effect under matched budget; gray annotations = Optuna budget effect. DL meta contributes +0.105 (HCC)/+0.083 (Multi), while budget upgrade contributes only +0.0005/+0.004.

To quantify the contribution of the DL metafeature, we conducted an ablation study comparing ML models trained on the 421-dimensional feature vector (AAC + DPC + length, without the DL metafeature) with models trained on the full 422-dimensional vector (with the DL metafeature) (Table 3, Figure 5). Removing the DL metafeature caused a substantial AUROC drop of −0.105 on HCC (0.883 → 0.778) and −0.087 on Multi (0.913 → 0.826) consistently across all three ML models. This demonstrates that the single DL metafeature dimension is the dominant signal in the ML framework, contributing +8.7–10.5 pp in AUROC. Notably, the contribution of the DL metafeature was larger for HCC (+0.105) than for Multi (+0.087), suggesting that the transformer signal has greater marginal value when the training data are limited. The sequence-only baseline (421-dim) achieved an AUROC of 0.778–0.826, indicating that AAC and DPC features alone provide a moderate but insufficient signal for Kla prediction. A complementary feature-group ablation (Table S6) and a SHAP analysis (Figure S3) corroborate this conclusion: the single 1-dim DL metafeature alone reproduces almost the full performance, and it accounts for the largest share of the model’s mean |SHAP| value (69.7% on HCC, 43.6% on Multi), whereas the amino-acid- and dipeptide-composition features contribute comparatively little.

To address potential confounding between feature-dimension change and Optuna budget difference, we performed an additional 422-dimensional ablation under the matched lite Optuna budget (10 trials × 3-fold CV; Table 3, ‘Lite’ rows). The 422-dim Lite Ensemble achieved AUROC 0.8826 on HCC (vs. 0.8831 Full; Δ = −0.0005) and 0.9086 on Multi (vs. 0.9126 Full; Δ = −0.004), demonstrating that increasing the Optuna budget from lite to full contributed only marginal improvement. Under the matched lite budget, the 421-dim → 422-dim transition still yielded +0.105 (HCC) and +0.083 (Multi) AUROC, virtually identical to the gains observed under unequal budgets. Thus, the DL meta-feature effect was decoupled from tuning-budget asymmetry and is approximately 20–210× larger than the Optuna budget effect, confirming the DL meta-feature as the dominant signal in the ML framework.

### 2.8. Naive Versus Proper Metafeature Generation

We systematically compared metafeature generation strategies in the pipeline (Table 4). In the naive approach, training metafeatures were generated from direct in-fold DL predictions, creating a distribution mismatch with the test metafeatures from unseen data. In the proper approach (out-of-fold metafeature generation [30]), training metafeatures were generated via 5-fold OOF predictions and test metafeatures via 5-fold averaged predictions, ensuring distributional consistency.

Switching from naive to proper metafeature generation recovered +20.3 pp for LightGBM and +26.5 pp for XGBoost. After correction, the individual ML models converged to the DL-only baseline (0.8827), confirming effective metafeature transfer. The ensemble then captured complementary signals for marginal additional improvements.

### 2.9. Feature Space Analysis via t-SNE

**Figure 6:**
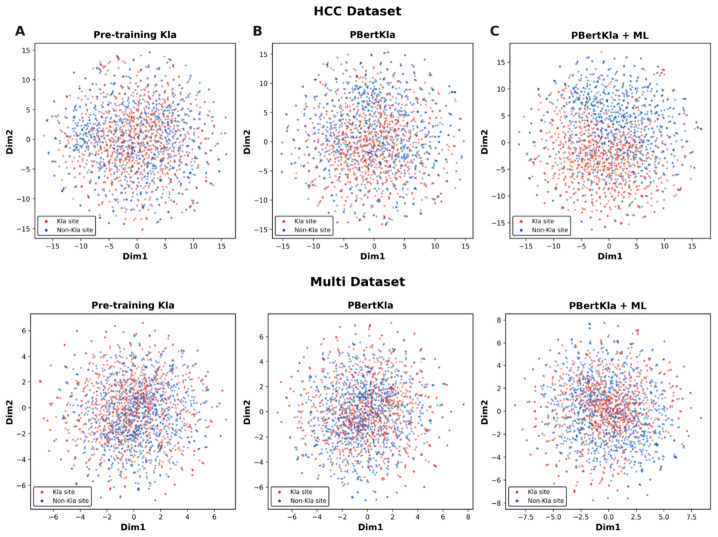
t-SNE visualization of HCC (**top**) and Multi (**bottom**) datasets. (**A**) Pretraining Kla (421-dim), (**B**) PBertKla (422-dim, +DL meta), and (**C**) PBertKla + ML (426-dim, +ML predictions). Red = Kla sites, and blue = non-Kla sites. Progressive class separation improvement across stages.

The feature space changes across the pipeline stages were visualized using t-SNE (Figure 6). The three stages differ in input dimensionality: (A) Pretraining Kla (421-dim: AAC 20 + DPC 400 + length 1)—raw sequence statistics without model training; (B) PBertKla (422-dim: + Transformer OOF prediction 1)—DL-learned signal added; and (C) PBertKla + ML (426-dim: + LightGBM/XGBoost/CatBoost/Ensemble predictions 4)—combined DL + ML signal.

In both the HCC and Multi datasets, a visually observable trend toward improved class separation between Kla sites (red) and non-Kla sites (blue) was observed across the stages. In the pretraining stage, the two classes were largely indistinguishable, whereas DL metafeature addition (PBertKla stage) initiated separation, and ML prediction addition (PBertKla + ML stage) further enhanced it. This qualitative trend is corroborated by quantitative class-separation metrics—the silhouette coefficient and the Davies–Bouldin index computed across the three pipeline stages (Figure S5; Table S9): separation improves modestly at each stage, but the low absolute silhouette values indicate that the Kla and non-Kla classes still overlap substantially.

### 2.10. Exploratory Structural Case Study: FAM210A

We selected FAM210A (UniProt Q96ND0) for this exploratory case study for three reasons. First, mitochondrial lysine lactylation has recently emerged as a key node of metabolic adaptation: AARS2 was identified as a hypoxia-responsive mitochondrial lactyltransferase that lactylates mitochondrial proteins such as PDHA1 and CPT2 to suppress glucose and fatty-acid oxidation and limit oxidative phosphorylation under hypoxia [31]. FAM210A is a mitochondrial regulator whose loss reprograms the tricarboxylic acid (TCA) cycle toward acetyl-CoA accumulation and protein hyperacetylation [32], linking it to lactate-derived lysine acylation; yet, its lactylation sites have not been reported to date, making it an attractive and under-explored candidate for in silico Kla-site prioritization within this pathway. Second, it is a genetically validated determinant of muscle and bone strength [33], giving it broad physiological relevance. Third, being absent from our training data, it provides a genuine out-of-sample illustration whose 26 lysines span both confident (helical) and intrinsically disordered regions.

We applied all four models (PBertKla HCC, PBertKla Multi, PBertKla + ML HCC, and PBertKla + ML Multi) to predict Kla for all 26 lysine sites in FAM210A (UniProt: Q96ND0, 272 residues). Sites predicted as Kla by ≥3 of the four models were classified as majority-vote Kla. Full 4-model prediction results are presented in Supplementary Table S5.

Twelve sites were classified as Kla (positive count ≥ 3: K87, K108, K109, K118, K214, K246, K251, K257, K262, K264, K269, and K270), six as non-Kla (positive count = 0: K33, K53, K84, K135, K158, and K180), and eight as borderline (positive count 1–2). Nine sites (K87, K108, K118, K214, K246, K251, K257, K262, and K264) showed unanimous agreement across all four models.

**Figure 7:**
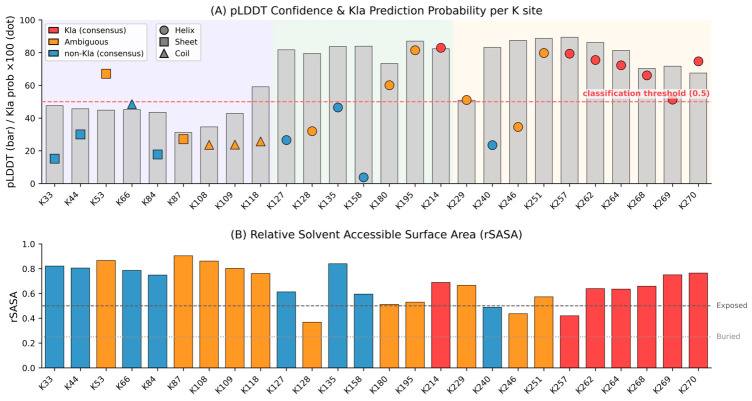
Structural analysis of 26 lysine sites in FAM210A. (**A**) Per-residue pLDDT (gray bars) and Kla prediction probability (colored dots); marker shape denotes secondary structure (circle = helix, square = sheet, triangle = coil) and color denotes consensus class. (**B**) Relative solvent-accessible surface area (rSASA) per lysine. The red dashed line in (**A**) indicates the classification threshold (probability = 0.5). In (**B**), the top dashed line indicates the exposed boundary (rSASA = 0.5) and the bottom dotted line indicates the buried threshold (rSASA = 0.2).

Structural analysis using the AlphaFold2 model (AF-Q96ND0-F1; AlphaFold DB model version v6) revealed that 8 of 12 majority-vote Kla sites (67%) were located in α-helices versus 50% of non-Kla sites—a higher but statistically non-significant proportion (Fisher’s exact *p* = 0.63; Table 5, Figure 7).

We present the FAM210A structural distribution purely as an exploratory illustration and make no claim of a quantitative relationship between AlphaFold confidence (pLDDT) and predicted Kla probability. The group comparisons (helix enrichment by Fisher’s exact test, SASA by Mann–Whitney U) did not reach significance.

**Figure 8:**
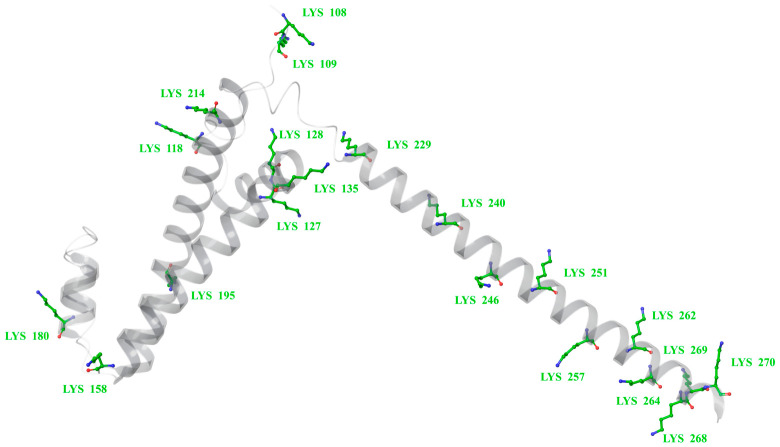
AlphaFold-predicted structure of FAM210A. Gray cartoon with green ball-and-stick representation of all 26 lysine side chains. Majority-vote Kla sites concentrated in core and C-terminal helical regions. Atom colors follow standard CPK convention (red = oxygen, blue = nitrogen).

FAM210A can be divided into three structural regions (Figure 8): (i) N-terminal disordered region (K33–K84; pLDDT 31–48; Sheet/Coil) → predominantly non-Kla; (ii) core helical bundle (K87–K214; mixed pLDDT) → mixed Kla/non-Kla; (iii) C-terminal repeat helix (K229–K270; pLDDT 51–89; Helix) → predominantly Kla. The near-identical Multi model probabilities (0.98–0.99) for C-terminal sites K257–K270 suggest possible sequence bias from the repeat motif (MEETKELITEKMEETK…), which is discussed as a limitation.

## 3. Discussion

Our results demonstrate that dataset expansion (HCC → Multi, AUROC +2.36 pp) yields greater performance improvement than architectural modification (the ML layer, +0.04–0.63 pp) in Kla prediction. The Multi dataset (26,034 samples) substantially exceeds existing Kla tool datasets (FSL-Kla ~2 K, DeepKla ~4 K, Auto-Kla ~5 K, and PBertKla ~10 K) and provides a quality-controlled community benchmark.

Decomposing the performance shows that the predictive signal derives primarily from training on the expanded dataset and from the ProteinBERT metafeature rather than from the ML layer per se: removing the metafeature costs 8.7–10.5 pp AUROC, whereas the ML layer adds a smaller increment (+0.04–0.63 pp; non-significant on HCC). We therefore do not claim a large accuracy jump from the ML layer; rather, the value of PBertKla + ML is that, trained on the integrated dataset, it reaches parity with the strongest available tool (Auto-Kla) on a common blind test set while remaining a practical, modular implementation—the heavy ProteinBERT backbone is decoupled from a lightweight ML head, so additional sequence- or structure-derived descriptors can be incorporated by retraining only that head. This modularity contrasts with the end-to-end feature fusion of PCBert-Kla [16], which requires full retraining to incorporate new features. Meta-classifier ensembles have precedent in related PTM-site prediction (e.g., STALLION for lysine acetylation [34]). The curated benchmark and its leakage-controlled validation (Section 2.5) provide the integrated training data and the evidence of robustness that make this result trustworthy. Although lysine lactylation is increasingly implicated in cancer metabolism, immune regulation, and fibrosis [6,7,8], we make no direct drug-discovery claims: the predictions are computational hypotheses for prioritizing candidate sites and require experimental validation.

The ablation study (Table 3, Figure 5) unambiguously demonstrates that the DL metafeature is the dominant signal in the ML framework, contributing +8.7–10.5 pp in AUROC across all datasets and models. Without it, the 421-dimensional sequence-only features (AAC + DPC + length) achieved AUROC of 0.778 on HCC and 0.826 on Multi, which were substantially below the DL-only baseline (0.883 and 0.906, respectively). This confirms that the modular ML design serves a clear purpose; it provides a structured channel through which the transformer-learned signal is combined with classical sequence statistics to achieve the final prediction. The larger DL metafeature contribution on HCC (+0.105) versus Multi (+0.087) further suggests that the transformer signal has a greater marginal value when the dataset is smaller, and the sequence statistics alone are less informative.

We further verified that the ablation gain is not driven by Optuna budget asymmetry. By repeating the 422-dim training under the matched lite Optuna budget (10 trials × 3-fold CV) and observing only −0.0005 (HCC) and −0.004 (Multi) AUROC change relative to the full budget, we established that the DL meta-feature contributes 20–210× more than the budget effect. This 2 × 2 controlled comparison (421 Lite, 422 Lite, 422 Full) ensures that the +8.3–10.5 pp budget-matched ablation gain reflects genuine ProteinBERT-derived signal rather than confounded hyperparameter optimization.

The naive versus proper metafeature-generation comparison provides a practical lesson: naive metafeature generation caused a +20–27 pp performance degradation owing to train-test distribution mismatch. This finding is relevant to practitioners building metafeature-based models for PTM prediction for the first time.

In the FAM210A case study, the higher (though non-significant; Fisher’s exact *p* = 0.63) helix proportion among majority-vote Kla sites (67% versus 50%) was directionally consistent with the findings of Li et al. [35], who reported helix enrichment among acetylated lysines in the human proteome. Because p300/CBP catalyzes both Kac and Kla [1,5], shared structural preferences are plausible. However, the same study reported a coil preference for KAT-specific acetylation [35], with caution against simplistic conclusions. The FAM210A case study is limited to a single protein (*n* = 26 K) and is presented as an exploratory illustration only; we do not interpret its structural distribution as evidence of a quantitative structure–probability relationship.

Limitations: (i) structural analysis is limited to FAM210A; (ii) the predictions are computational hypotheses and no experimental (wet-lab) validation has been performed; (iii) C-terminal repeat sequence bias is possible; (iv) the ML layer’s AUROC gain over PBertKla on HCC is +0.0004, effectively negligible; and (v) DeepKla could not be re-evaluated on our blind test set because its released implementation is frozen at end-of-life Python 2.7/Keras and does not run on current frameworks, so it is compared only via its reported performance (Auto-Kla and PCBert-Kla were re-trained on our split and compared directly in Section 2.6). Future work will include ESM2 embedding integration, proteome-wide AlphaFold secondary structure analysis, and multitarget wet-lab validation.

## 4. Materials and Methods

### 4.1. Data Collection and Preprocessing

The HCC dataset was obtained from the original PBertKla study [15], which was derived from mass spectrometry-identified Kla sites in human hepatocellular carcinoma [15]. It contains 4820 positive and 4820 negative samples, which were split 80:20 into training (7712) and test (1928) sets.

The Multi dataset was assembled from nine published Kla studies covering human HCC [15], gastric cancer [20], human lung [21], HCC proteomics [22], rat [23], *Toxoplasma gondii* [24], *Phialophora verrucosa* [25], *Botrytis cinerea* [26], and rice (*Oryza sativa*) [27]. Raw data (16,223 positive/72,209 negative) underwent the 9-step quality-control pipeline described in Section 2.1, yielding 13,017 positive and 13,017 negative samples split into training (20,827) and test (5207) sets.

Sequence representation used a 45-residue window centered on the target lysine (±22 residues). Non-standard amino acids are denoted ‘X’; windows containing ≥5 ‘X’ characters were removed due to insufficient information.

### 4.2. Stage 1: ProteinBERT Fine-Tuning

ProteinBERT [28] is a protein language model pretrained on 23.5 M sequences from UniRef90. Its architecture comprises a dual-path structure with six transformer blocks, including a local path for position-specific representations and a global path for gene ontology-based global representations, incorporating wide and narrow conv-1D, add and norm, dense, and global attention layers. We used the checkpoint at epoch 92,400/sample 23.5 M (191 MB).

Fine-tuning was performed with 5-fold StratifiedKFold (seed = 42). Each fold followed three phases: (i) frozen warmup—6-block encoder weights frozen, classification head (Dense + Sigmoid) trained for initial stabilization; (ii) full fine-tuning—all parameters trained with lr = 2 × 10^−3^, batch size 32, input length 512 tokens, max 100 epochs, early stopping patience 15; and (iii) long-sequence fine-tuning—input length extended to 1024 tokens for one additional epoch to adapt positional encoding to longer contexts.

Metafeature generation: For each training sample, the OOF prediction from the fold, excluding the sample, served as the DL metafeature. For the test samples, the arithmetic mean of the predictions from all 5-fold models was used. This canonical out-of-fold metafeature approach [30] ensures distributional consistency between the training and test metafeatures.

The training was conducted on an NIPA H200 GPU server using a TensorFlow-based ProteinBERT (version 1.0.1) implementation with wandb logging.

### 4.3. Stage 2: ML Meta-Classifier

A 422-dimensional feature vector was constructed using AAC (20-dim: frequency of each standard amino acid), DPC (400-dim: frequency of each dipeptide), sequence length (1-dim), and DL metafeature (1-dim: OOF probability from Stage 1). The features were computed using a 45-residue window sequence.

Three gradient boosting models (LightGBM, XGBoost, and CatBoost) were optimized using Optuna [29] with 100 trials × 5-fold CV (seed = 42) to maximize the AUC-ROC. The search spaces included learning_rate, max_depth, n_estimators, and subsample. The optimal parameters are listed in Supplementary Table S4.

For the ablation fairness control reported in Section 2.7, we additionally trained 422-dimensional models under the matched lite Optuna budget (10 trials × 3-fold CV) on both HCC and Multi datasets, using the same run_ML.py script with reduced trial and CV arguments.

### 4.4. Stage 3: Soft-Voting Ensemble

The test predictions from LightGBM, XGBoost, and CatBoost were averaged as follows: p_final = (p_LGB + p_XGB + p_CAT)/3. A threshold of 0.5 was applied for binary classification. Weighted voting was tested but showed no significant advantage over uniform averaging.

### 4.5. t-SNE Visualization

The feature space changes across the pipeline stages were visualized using t-SNE. The test set feature vectors were constructed in three stages: pretraining Kla (421-dim), PBertKla (422-dim), and PBertKla + ML (426-dim). Scikit-learn’s t-SNE implementation (perplexity = 30, n_iter = 1000, random_state = 42) was used for 2D projections on both HCC and Multi datasets.

### 4.6. AlphaFold Structure Analysis

The AlphaFold2 [36] predicted structure of FAM210A (UniProt: Q96ND0; AF-Q96ND0-F1; AlphaFold DB model version v6) was downloaded from the AlphaFold Protein Structure Database [37]; the same region was additionally re-predicted with AlphaFold3 [38] for comparison (Figure S6). The structural features were computed as follows:

- pLDDT: Extracted from the B-factor column of the PDB file (BioPython 1.87 PDBParser).
- SASA: Computed using FreeSASA 2.2.1 [39] with the Lee-Richards algorithm (probe radius 1.4 Å, default). Relative SASA (rSASA) was normalized by the Gly-X-Gly maximum SASA for lysine (230.0 Å^2^; Tien et al. [40]).
- Secondary structure: Assigned using biotite 1.6.0 [41] annotate_sse function (α-helix, β-sheet, and coil).
- Statistical tests: Mann–Whitney U test (Kla versus non-Kla group comparisons), and Fisher’s exact test (2 × 2 contingency table for helix versus non-helix × Kla versus non-Kla). All tests used SciPy.
- Three-dimensional visualization: PyMOL 3.1.0 with cartoon representation (pLDDT-based spectrum coloring), lysine side-chain ball-and-stick, and ray-traced rendering (2400 × 1800 px).

The per-residue structural features (pLDDT, SASA, rSASA, secondary structure, and majority-vote classification) for all 26 lysines of FAM210A are provided in Table S10; the analysis script (analyze_fam210a.py) is available in the project repository.

### 4.7. Evaluation Metrics

All the classification performances were evaluated using independent test sets. The reported metrics included AUROC, F1 score (macro average), accuracy, Matthews correlation coefficient (MCC), and area under the precision–recall curve (AUPRC). A default threshold of 0.5 was used.

### 4.8. Software and Hardware

DL training: NIPA H200 GPU (NVIDIA, Santa Clara, CA, USA), Python 3.8.20, TensorFlow 2.12.0, ProteinBERT 1.0.1, and Wandb 0.12.21. ML training: Python 3.10, LightGBM 4.3.0, XGBoost 2.0.3, CatBoost 1.2.5, and Optuna 3.6.1. Structure analysis: macOS, BioPython 1.87, FreeSASA 2.2.1, biotite 1.6.0, SciPy 1.10.1, matplotlib 3.10.3, and PyMOL 3.1.0 (Schrödinger, LLC, New York, NY, USA). t-SNE: scikit-learn 1.2.2. All code and trained model weights are available at https://github.com/LNPSolution-dev/PBertKla-Machinelearning-stack, accessed on 19 June 2026.

### 4.9. Leakage-Controlled Splits

Leakage-controlled splits (Section 2.5) were constructed as follows. Protein-level: GroupShuffleSplit (test_size = 0.2, seed = 42) on UniProt accession, reconstructed for 21,113/26,034 windows (81.1%; 3594 unique proteins); windows without a recoverable accession were assigned to singleton groups. Homology-reduced: all unique windows were clustered with CD-HIT (-c 0.4 -n 2 -d 0) and whole clusters assigned to train/test. Leave-one-study-out: seven folds, each holding out one source study. The seven folds correspond to the seven of the nine source studies whose Kla sites map to UniProt entries; the two non-model-organism datasets (*Toxoplasma gondii* and *Phialophora verrucosa*) contained no UniProt-mappable sites and were removed at the early quality-control step requiring a known accession. For every split the pipeline (ProteinBERT 5-fold fine-tuning, OOF metafeature generation, and ML meta-classifier) was re-trained from scratch on the training side only; no test-side information entered fine-tuning, metafeature generation, or hyperparameter selection. Train–test overlap was verified to be zero by exact set intersection of the grouping key in every split.

## 5. Conclusions

In this work, we trained the existing PBertKla predictor on an integrated multi-source dataset and augmented it with a lightweight machine-learning classifier over sequence-derived features; on a common blind test set, the resulting PBertKla + ML model reached AUROC 0.9126 on the integrated set, was statistically indistinguishable from the strongest available tool (Auto-Kla), and exceeded a recent ProtBert-based method (PCBert-Kla). Ablation and SHAP analyses showed that this performance originates primarily from training on the expanded dataset and the ProteinBERT metafeature rather than the ML layer itself, so we present the combined classifier as a practical, modular implementation that reaches parity with the best available tool rather than as a source of large accuracy gains. Underpinning the model, we assembled and released the largest curated Kla benchmark to date (Multi; 26,034 samples, 9-step quality control) and validated it under a rigorous leakage-controlled protocol—protein-level, homology-reduced, and leave-one-study-out re-training all maintained ≈0.90 AUROC (within 1.5 pp of the random-split value)—demonstrating genuine generalization rather than train–test leakage. An exploratory AlphaFold-based case study of FAM210A illustrated how predicted Kla sites distribute across ordered and disordered regions; the structural analysis is presented as an exploratory observation rather than a quantitative structure–probability relationship. All code and model weights are publicly released to enable reproducible Kla prediction.

## Figures and Tables

**Table 1:** Dataset summary.

Dataset	Description	Positive	Negative	Total	Train	Test
HCC	Human HCC Kla	4820	4820	9640	7712	1928
Multi	Multispecies (9 sources)	13,017	13,017	26,034	20,827	5207

**Table 2:** Performance comparison across datasets and models (independent test sets). PBertKla results show 5-fold ensemble predictions with fold-level standard deviation in parentheses. PBertKla + ML results are single test-set evaluations of the final ensemble.

Dataset	Test *n*	Model	AUROC	F1 Score	ACC	MCC
HCC	1928	PBertKla	0.8827 (±0.0042)	0.7957 (±0.0086)	0.7946 (±0.0056)	0.589
Multi	5207	PBertKla	0.9063 (±0.0219)	0.8234 (±0.0083)	0.8160 (±0.0101)	0.634
HCC	1928	PBertKla + ML	0.8831	0.8111	0.8055	0.612
Multi	5207	PBertKla + ML	0.9126	0.8314	0.8245	0.651

**Table 3:** Ablation study with fairness control: effect of DL meta-feature under matched and full Optuna budgets. Under matched lite budget (10 trials, 3-fold CV), DL meta-feature addition yields +0.105 AUROC on HCC and +0.083 on Multi. Optuna budget upgrade alone contributes only +0.0005 (HCC) and +0.004 (Multi).

Dataset	Model	Feature	Optuna	AUROC	F1	ACC	ΔAUROC
HCC	Ensemble	421-dim	Lite (10 × 3)	0.778	0.715	0.703	—
HCC	Ensemble	422-dim	Lite (10 × 3)	0.883	0.804	0.800	+0.105
HCC	Ensemble	422-dim	Full (100 × 5)	0.883	0.811	0.806	+0.105
Multi	Ensemble	421-dim	Lite (10 × 3)	0.826	0.752	0.747	—
Multi	Ensemble	422-dim	Lite (10 × 3)	0.909	0.825	0.818	+0.083
Multi	Ensemble	422-dim	Full (100 × 5)	0.913	0.831	0.825	+0.087

**Table 4:** Naive vs. proper metafeature-generation comparison (HCC dataset, AUROC).

Model	Naive	Proper	Δ
LightGBM	0.6798	0.8825	+0.2027
XGBoost	0.6183	0.8832	+0.2649
Ensemble	0.7332	0.8831	+0.1499

**Table 5:** FAM210A structural properties: majority-vote Kla vs. non-Kla (4-model majority vote).

Property	Kla (*n* = 12)	non-Kla (*n* = 6)	Test	*p*
pLDDT	68.5 ± 20.8	62.9 ± 17.9	Mann–Whitney	0.682
SASA (Å^2^)	157.9 ± 33.7	168.0 ± 30.5	Mann–Whitney	0.617
α-helix	8/12 (67%)	3/6 (50%)	Fisher’s exact	0.627

## Data Availability

Datasets, trained model weights, and analysis scripts are available at https://github.com/LNPSolution-dev/PBertKla-Machinelearning-stack, accessed on 19 June 2026. The FAM210A AlphaFold structure is accessible from the AlphaFold Protein Structure Database (UniProt: Q96ND0).

## Supplementary Materials

The following supporting information can be downloaded at: https://www.mdpi.com/article/10.3390/ijms27135761/s1.
