# Supplementary material for: Training PBertKla on an Integrated Multi-Source Dataset with a Machine-Learning Layer for Lysine Lactylation Site Prediction
Source: Int J Mol Sci. 2026 Jun 26;27(13):5761. doi: 10.3390/ijms27135761 (PMC13362394; doi:10.3390/ijms27135761)
Supplement: Supplementary file 1 [file ijms-27-05761-s001.zip › ijms-4330372-supplementary.pdf]

International Journal of Molecular Sciences

MDPI

Supplementary Materials

Training PBertKla on an Integrated Multi-Source Dataset with a Machine-Learning Layer for Lysine Lactylation Site Prediction

Table S1

Table S1. 9-step quality control (QC) pipeline applied to the multi-species dataset. Kla denotes lysine lactylation; non-Kla denotes non-lactylated lysine residues. The "remaining" columns indicate the number of sites retained after each step.

Step	Description	Kla remaining	Non-Kla remaining
Raw	Source data collection	19,245	72,209
1	Remove unknown UniProt ID (empty sequence)	16,222	
2	Remove non-K site entries	16,021	—
3	Remove sites exceeding sequence length	16,009	—
4	Add PBertKla opensource data	18,295	—
5	Remove duplicate entries	13,017	
6	Remove K data outside ±45 range	—	19,137
7	Remove proximal K duplicates + X≥5	—	17,656
8	Remove non-Kla duplicates	—	18,768
9	1:1 class balancing	13,017	13,017

Table S2 & Figure S1

Table S2a. Five-fold cross-validation results of the PBertKla model on the HCC dataset. ACC, accuracy; Prec, precision; F1, F1-score; AUROC, area under the receiver operating characteristic curve; AUPRC, area under the precision–recall curve; avg, average across the five folds.

Fold	ACC	Prec	Recall	F1	AUROC	AUPRC
1	0.787	0.784	0.793	0.788	0.862	0.839
2	0.782	0.783	0.779	0.781	0.874	0.859
3	0.785	0.781	0.793	0.787	0.870	0.845
4	0.775	0.781	0.766	0.773	0.868	0.850
5	0.775	0.791	0.747	0.768	0.867	0.852
avg	0.795	0.792	0.800	0.796	0.883	0.863

Table S2b. Five-fold cross-validation results of the PBertKla model on the multi-species (Multi) dataset. Abbreviations as in Table S2a.

Fold	ACC	Prec	Recall	F1	AUROC	AUPRC
1	0.817	0.801	0.843	0.821	0.900	0.897
2	0.820	0.800	0.855	0.826	0.906	0.904

3	0.816	0.790	0.860	0.824	0.901	0.897
4	0.795	0.766	0.850	0.806	0.853	0.796
5	0.808	0.788	0.843	0.814	0.898	0.894
avg	0.816	0.791	0.858	0.823	0.906	0.904

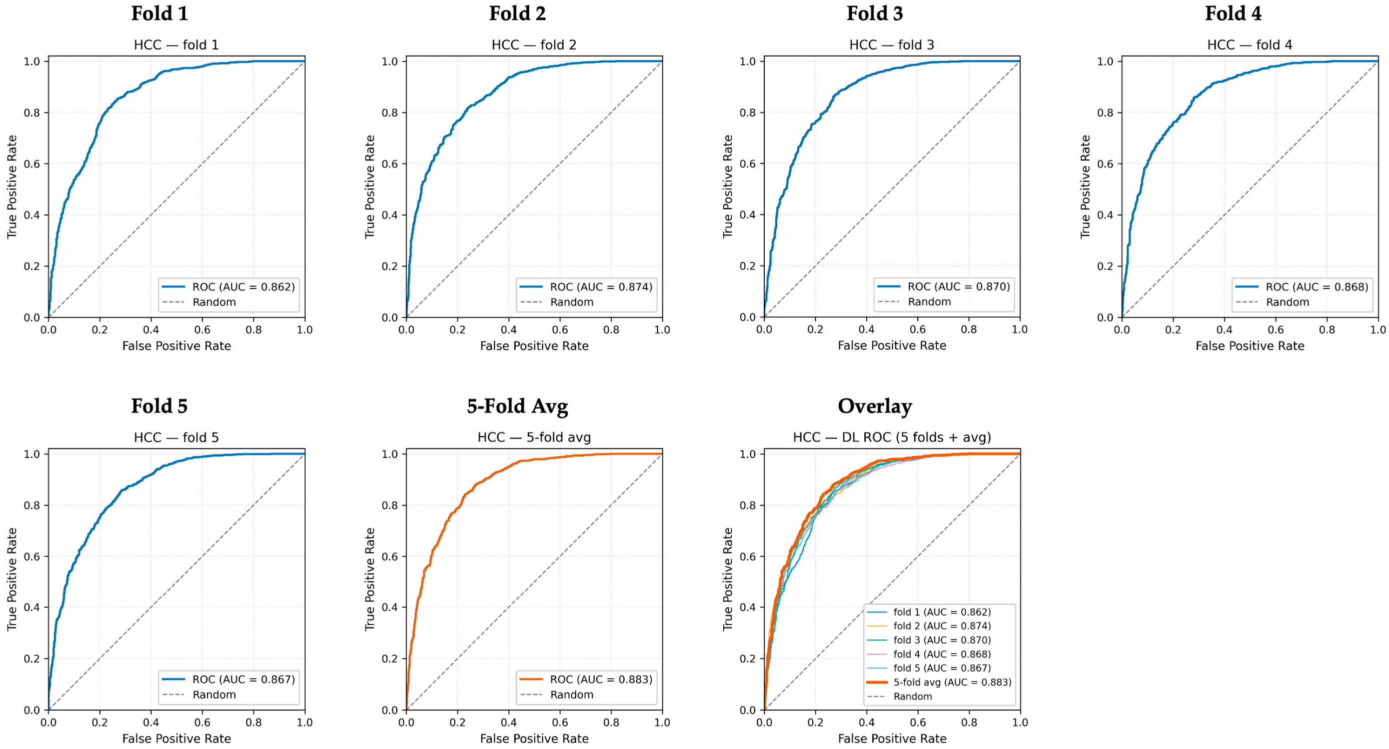

Figure S1a. Receiver operating characteristic (ROC) curves for the PBertKla model on the HCC dataset, shown for each of the five cross-validation folds together with the average overlay across all folds.

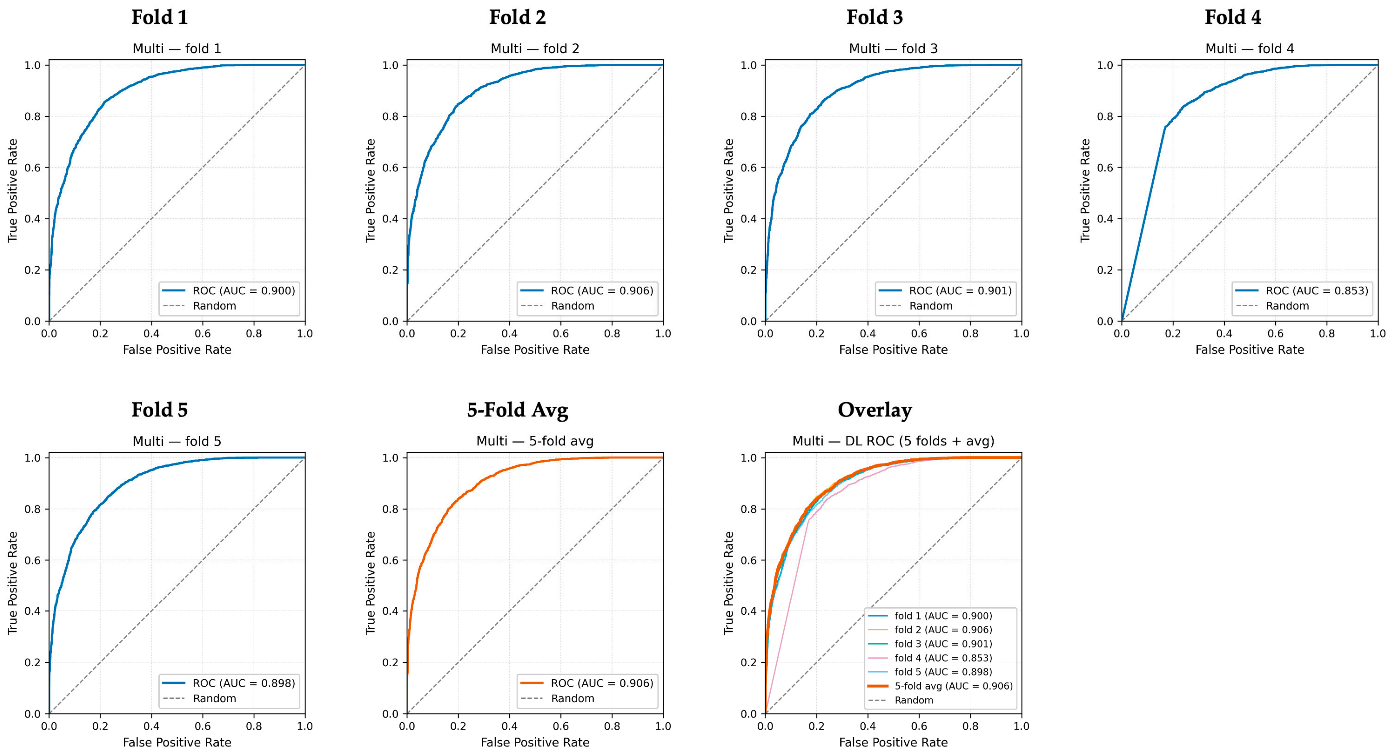

Figure S1b. ROC curves for the PBertKla model on the multi-species (Multi) dataset, shown for each of the five cross-validation folds together with the average overlay across all folds.

Table S3 & Figure S2

Table S3a. Performance of the individual machine learning (ML) classifiers and the ML meta-classifier within the PBertKla + ML pipeline on the HCC dataset. Abbreviations for the metric columns are as defined in Table S2a.

**Model**	**ACC**	**Prec**	**Recall**	**F1**	**AUROC**	**AUPRC**
LightGBM	0.805	0.789	0.832	0.810	0.883	0.862
XGBoost	0.805	0.788	0.833	0.810	0.883	0.863
CatBoost	0.804	0.787	0.834	0.810	0.883	0.862
Ensemble	0.806	0.788	0.835	0.811	0.883	0.862

Table S3b. Performance of the individual ML classifiers and the ML meta-classifier within the PBertKla + ML pipeline on the multi-species (Multi) dataset. Abbreviations as in Table S2a.

**Model**	**ACC**	**Prec**	**Recall**	**F1**	**AUROC**	**AUPRC**
LightGBM	0.825	0.803	0.863	0.832	0.910	0.910
XGBoost	0.821	0.797	0.863	0.828	0.911	0.910
CatBoost	0.823	0.798	0.865	0.830	0.913	0.913
Ensemble	0.825	0.800	0.866	0.831	0.913	0.912

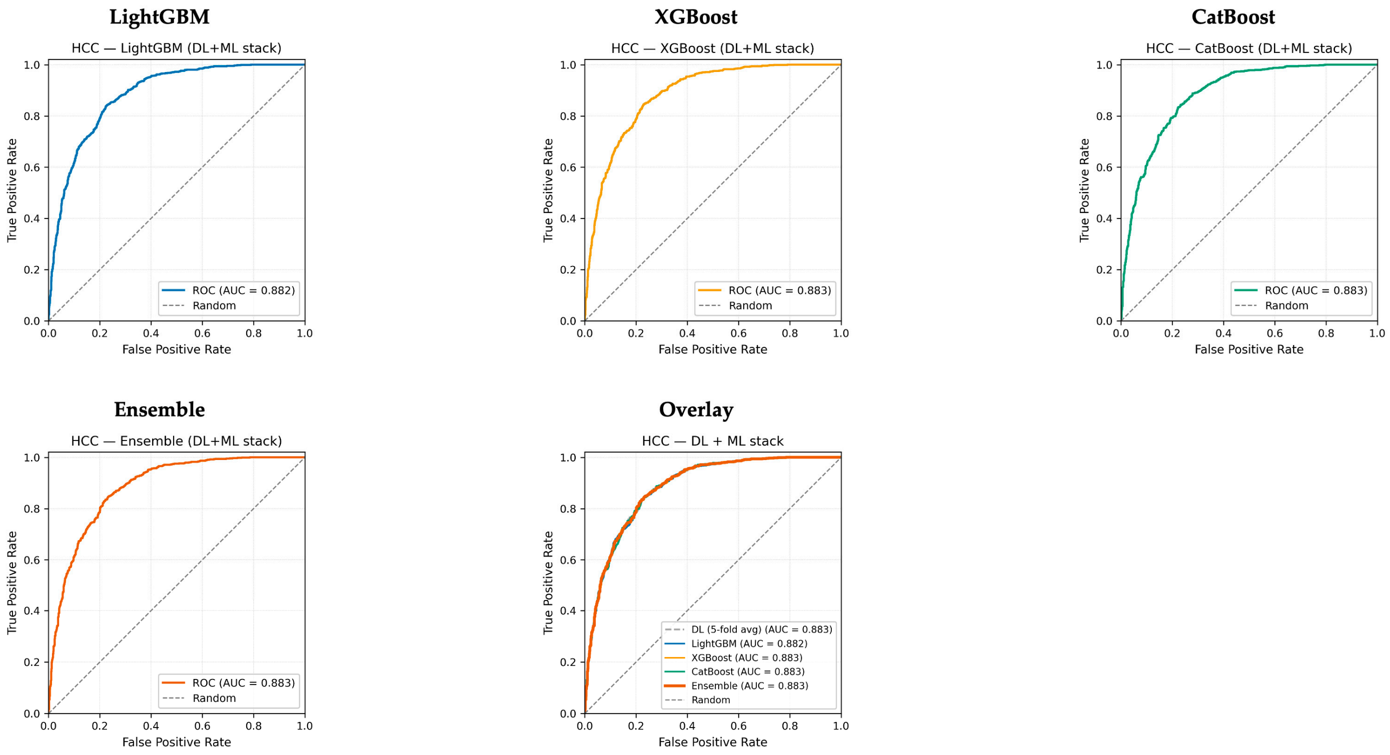

Figure S2a. ROC curves of the individual ML classifiers and the ML meta-classifier of the PBertKla + ML pipeline on the HCC dataset, together with the average overlay across all models.

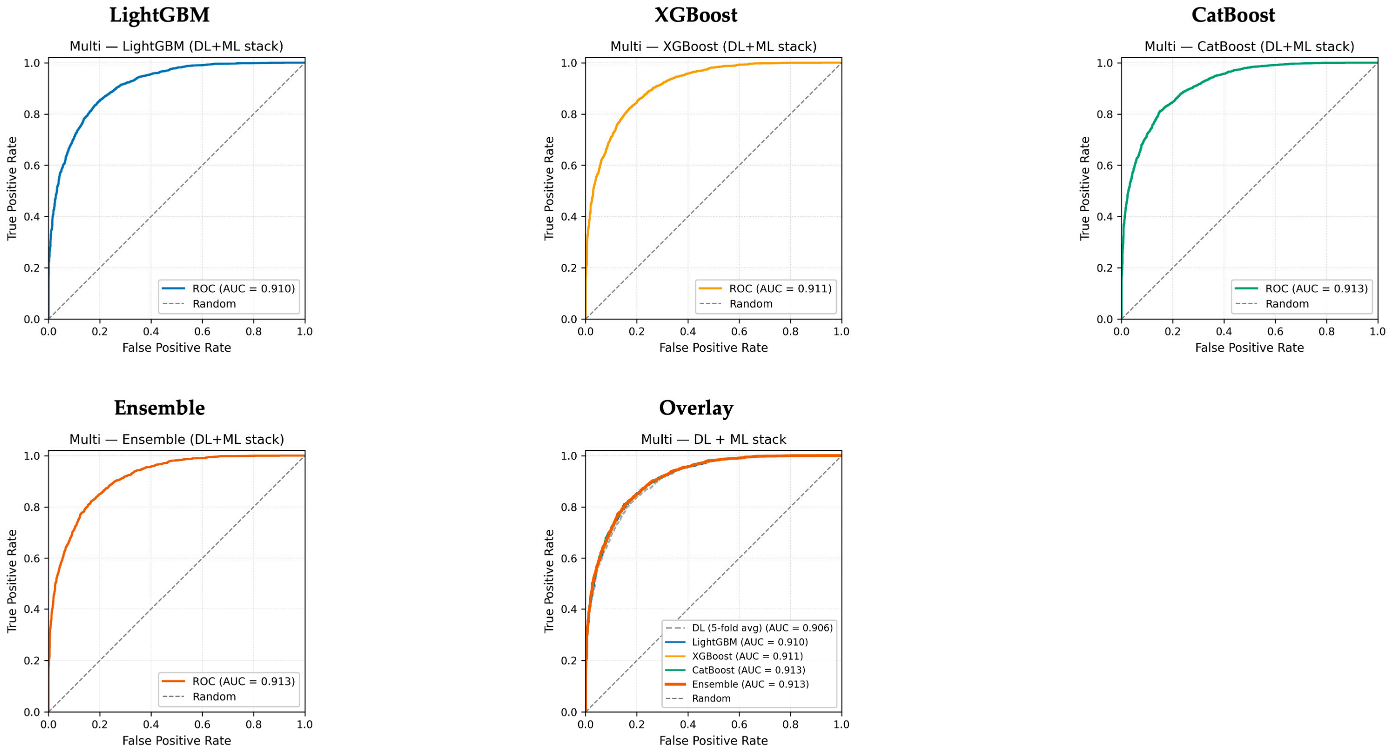

Figure S2b. ROC curves of the individual ML classifiers and the ML meta-classifier of the PBertKla + ML pipeline on the multi-species (Multi) dataset, together with the average overlay across all models.

Table S4

Hyperparameters were optimized using the Optuna Tree-structured Parzen Estimator (TPE) sampler with 100 trials and 5-fold cross-validation (seed = 42), maximizing AUROC for the 422-dimensional ML meta-classifier configuration.

Table S4a. Optimized LightGBM hyperparameters for the HCC and multi-species (Multi) datasets.

Hyperparameter	Range	HCC	Multi
n_estimators	[100, 2000]	562	1,888
learning_rate	[0.005, 0.3]	0.00699	0.01423
max_depth	[3, 12]	3	11
num_leaves	[15, 255]	68	198
min_child_samples	[5, 100]	95	7
subsample	[0.5, 1.0]	0.660	0.698
colsample_bytree	[0.5, 1.0]	0.680	0.531
reg_alpha	[1e-8, 10]	1.123	0.190
reg_lambda	[1e-8, 10]	4.342	1.07e-8
Best CV AUC	—	0.8686	0.8889

Table S4b. Optimized XGBoost hyperparameters for the HCC and Multi datasets.

Hyperparameter	Range	HCC	Multi
n_estimators	[100, 2000]	320	1,529
learning_rate	[0.005, 0.3]	0.01099	0.00887
max_depth	[3, 10]	3	10
subsample	[0.5, 1.0]	0.866	0.638
colsample_bytree	[0.5, 1.0]	0.659	0.644
gamma	[1e-8, 5]	1.53e-5	0.807
reg_alpha	[1e-8, 10]	1.344	0.175
reg_lambda	[1e-8, 10]	0.127	2.616
min_child_weight	[1, 20]	13	1
Best CV AUC	—	0.8690	0.8895

Table S4c. Optimized CatBoost hyperparameters for the HCC and Multi datasets.

Hyperparameter	Range	HCC	Multi
iterations	[200, 3000]	414	2,902
learning_rate	[0.005, 0.3]	0.00564	0.01367
depth	[4, 10]	7	10
l2_leaf_reg	[1e-3, 10]	4.034	0.121
bagging_temperature	[0, 1]	0.320	0.116
border_count	[32, 255]	147	81
Best CV AUC	—	0.8691	0.8924

Table S5

Table S5. Predicted lactylation status of the 26 lysine residues of FAM210A by four prediction models at a probability threshold of 0.5. PB HCC and PB Multi denote the PBertKla model trained on the

HCC and multi-species datasets, respectively; PB+ML HCC and PB+ML Multi denote the corresponding PBertKla + ML ensemble models. “Kla” indicates a positive prediction (predicted to be lactylated) and “—” indicates a negative prediction. Pos counts the number of models (out of four) returning a positive prediction. The Vote column shows the majority-vote consensus: “Kla” if at least three of four models predict lactylation, “non” if no model predicts lactylation, and “—” otherwise.

K	PB HCC	PB Multi	PB+ML HCC	PB+ML Multi	Pos	Vote
K33	—	—	—	—	0	non
K44	—	—	Kla	—	1	—
K53	—	—	—	—	0	non
K66	—	Kla	—	—	1	—
K84	—	—	—	—	0	non
K87	Kla	Kla	Kla	Kla	4	Kla
K108	Kla	Kla	Kla	Kla	4	Kla
K109	—	Kla	Kla	Kla	3	Kla
K118	Kla	Kla	Kla	Kla	4	Kla
K127	—	Kla	—	Kla	2	—
K128	—	Kla	—	Kla	2	—
K135	—	—	—	—	0	non
K158	—	—	—	—	0	non
K180	—	—	—	—	0	non
K195	—	—	Kla	—	1	—
K214	Kla	Kla	Kla	Kla	4	Kla
K229	Kla	—	Kla	—	2	—
K240	Kla	—	Kla	—	2	—
K246	Kla	Kla	Kla	Kla	4	Kla
K251	Kla	Kla	Kla	Kla	4	Kla
K257	Kla	Kla	Kla	Kla	4	Kla
K262	Kla	Kla	Kla	Kla	4	Kla
K264	Kla	Kla	Kla	Kla	4	Kla
K268	—	Kla	—	Kla	2	—
K269	—	Kla	Kla	Kla	3	Kla
K270	—	Kla	Kla	Kla	3	Kla

Table S6

Feature set	# feat.	HCC AUROC	HCC F1	HCC ACC	Multi AUROC	Multi F1	Multi ACC
**Full (422-dim)**	**422**	**0.8721**	**0.7971**	**0.7941**	**0.9073**	**0.8300**	**0.8231**
DL_meta only (1-dim)	1	0.8767	0.8021	0.8008	0.9052	0.8236	0.8156
AAC+DPC+Length (421-dim, no DL)	421	0.7658	0.7014	0.6909	0.8159	0.7434	0.7384

AAC+DL_meta (21-dim)	21	0.8746	0.7932	0.7905	0.9041	0.8245	0.8168
DPC+DL_meta (401-dim)	401	0.8676	0.7877	0.7853	0.9081	0.8271	0.8202
AAC only (20-dim)	20	0.7619	0.7009	0.6919	0.8059	0.7287	0.7256
DPC only (400-dim)	400	0.7361	0.6834	0.6795	0.8047	0.7370	0.7369

Table S6. Feature-group ablation of the PBertKla + ML classifier (LightGBM meta-classifier) on the HCC and multi-species (Multi) test sets. Rows differ in the subset of the 422-dimensional feature vector used: amino-acid composition (AAC, 20-dim), dipeptide composition (DPC, 400-dim), sequence length (1-dim), and the ProteinBERT DL metafeature (1-dim). The single 1-dim DL metafeature alone (AUROC 0.8767 HCC, 0.9052 Multi) matches or slightly exceeds the full 422-dim model (0.8721, 0.9073), whereas removing it (AAC+DPC+length, 421-dim) lowers AUROC to 0.7658 (HCC) and 0.8159 (Multi). The ProteinBERT metafeature, not the sequence-composition features, carries the predictive signal.

Figure S3

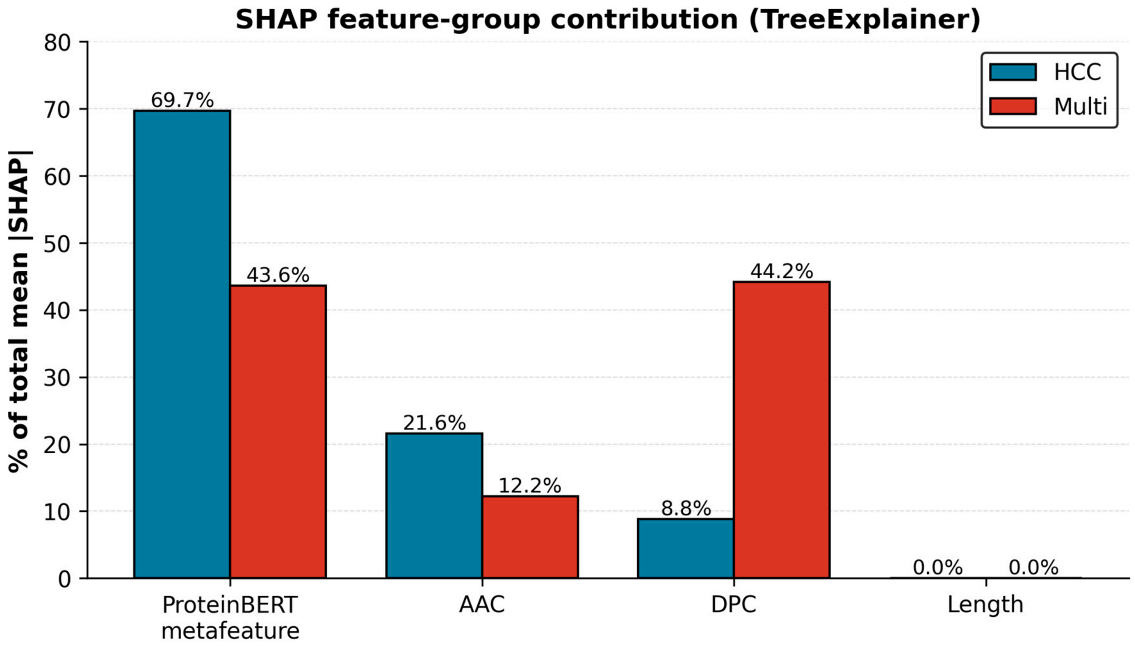

Figure S3. SHAP feature-group importance for the PBertKla + ML classifier (TreeExplainer), expressed as each group's percentage of the total mean |SHAP| value on the HCC and multi-species (Multi) test sets. The ProteinBERT DL metafeature accounts for the largest share (69.7% on HCC, 43.6% on Multi), far exceeding amino-acid composition (AAC), dipeptide composition (DPC), and sequence length, indicating that the ProteinBERT-derived signal dominates the model's predictions.

Figure S4

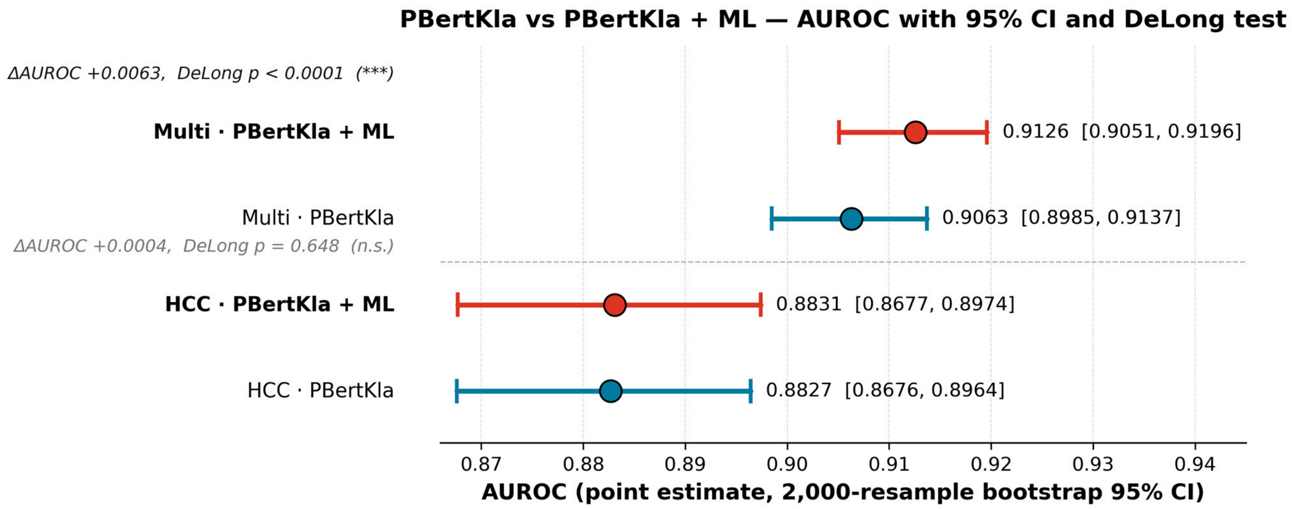

Figure S4. AUROC with bootstrap 95% confidence intervals and DeLong test for PBertKla versus PBertKla + ML on the HCC and multi-species (Multi) test sets. Points are AUROC point estimates; horizontal bars are 2,000-resample bootstrap 95% confidence intervals. The HCC difference is negligible and not statistically significant (ΔAUROC +0.0004; DeLong $p = 0.648$), whereas the Multi difference is small but statistically significant (ΔAUROC +0.0063; DeLong $p < 0.0001$).

Figure S5

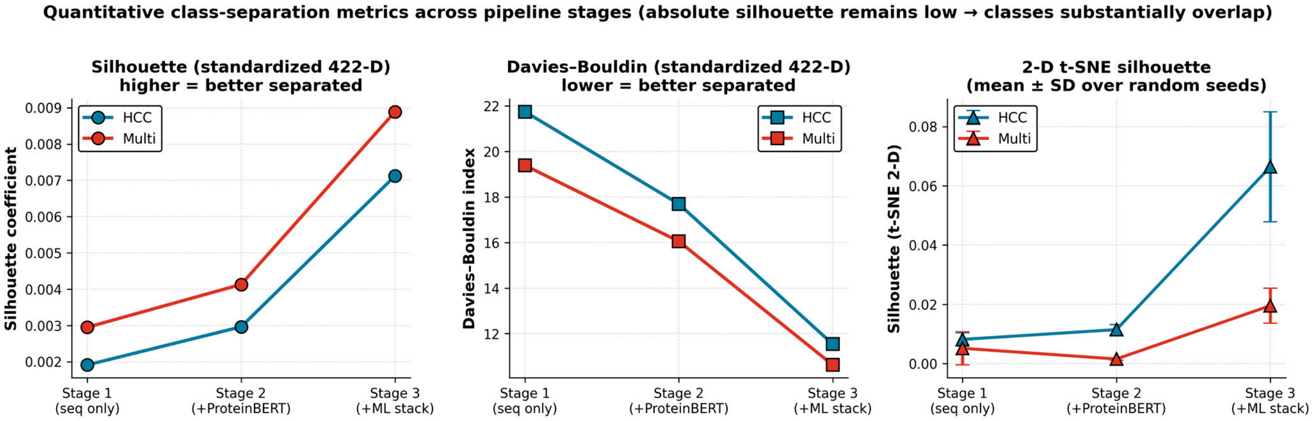

Figure S5. Quantitative class-separation metrics across the three pipeline stages (sequence-only → +ProteinBERT → +ML) on the HCC and multi-species (Multi) test sets. Left: silhouette coefficient on the standardized 422-dimensional feature space (higher = better separated). Middle: Davies–Bouldin index (lower = better separated). Right: silhouette coefficient of the 2-D t-SNE embedding (mean ± SD over random seeds). Separation improves modestly at each stage, but the low absolute silhouette values indicate that the Kla and non-Kla classes still overlap substantially.

Figure S6

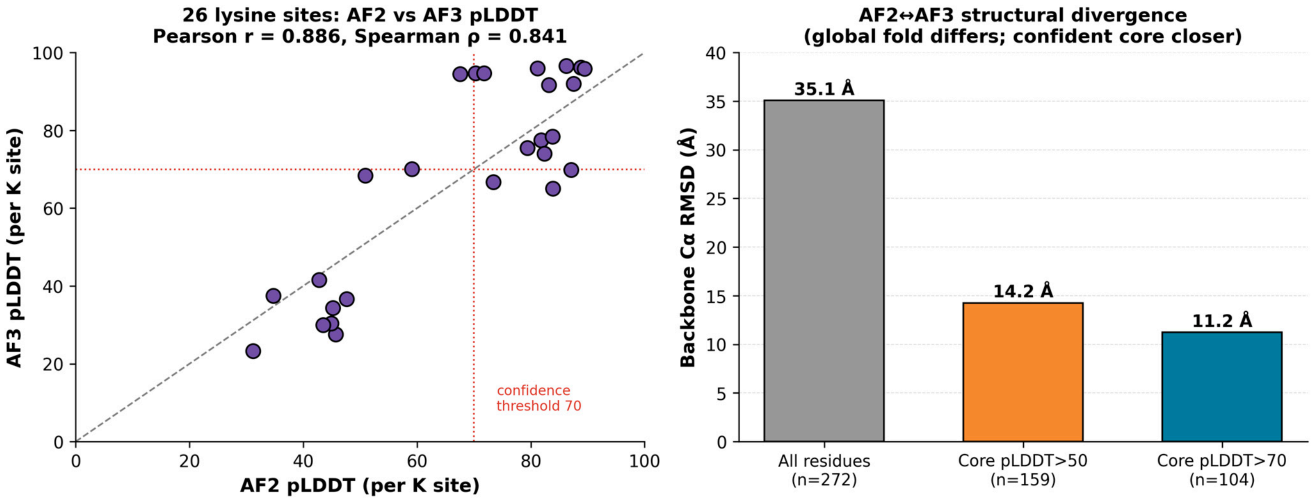

Figure S6. Comparison of the AlphaFold2 (AF2) and AlphaFold3 (AF3) predicted structures of FAM210A. Left: per-residue pLDDT of the 26 lysine sites, AF2 versus AF3 (Pearson r = 0.886, Spearman ϱ = 0.841); dotted lines mark the pLDDT = 70 confidence threshold. Right: backbone Cα RMSD between the two models for all residues (35.1 Å), the pLDDT > 50 core (14.2 Å), and the pLDDT > 70 confident core (11.2 Å). The two generations agree closely on per-residue confidence and assign all lysine sites low confidence, while the global fold differs.

Table S7

Table S7. Leakage-controlled re-training of the full pipeline on the Multi benchmark. The complete pipeline (ProteinBERT fine-tuning → out-of-fold metafeature → ML meta-classifier) was re-trained from scratch under each split; the exact set intersection of the grouping key between train and test was empty (leakage = 0) in every case. These values are summarized in Figure 3 of the main text.

Evaluation split	Leakage control	Ensemble AUROC	AUPRC
Random 80:20 (original)	none	0.9126	0.9122
Protein-level	GroupShuffleSplit by UniProt accession	0.8974	0.9036
Homology-reduced	CD-HIT 40% clustering, whole clusters to one side	0.9064	0.9041
Leave-one-study-out (7 folds)	each source study held out entirely	0.9034 (mean; range 0.866–0.942)	—

Table S8

Table S8. Head-to-head comparison of Kla predictors re-trained on the Multi training split and evaluated on the identical blind test set (n = 5,207), with full standard metrics. DeepKla is shown from its reported literature performance only, as its end-of-life Python 2.7 implementation cannot be faithfully re-run. These values are summarized in Figure 4 of the main text.

Method	Backbone	AUROC	AUPRC	ACC	F1	MCC	Note
PBertKla (reproduced) [9]	ProteinBERT	0.9063	0.9044	0.8160	0.8234	—	retrained, same test set

PBertKla + ML (this work)	Protein-BERT + ML	0.9126	0.9122	0.8245	0.8314	0.651	—
Auto-Kla [retrained, 8]	ELECTRA (AutoGluon)	0.9133	0.9098	0.8227	0.8356	0.654	retrained, same test set
PCBert-Kla [retrained, 10]	ProtBert (4-layer) + physico.	0.8941	0.8915	0.8051	0.8001	0.611	retrained, same test set
DeepKla [7]	CNN-BiGRU-Attn (Py2.7)	—	—	—	—	—	literature only (see text)

Table S9

Table S9. Quantitative class-separation metrics underlying Figure S5, across the three pipeline stages (sequence-only → +ProteinBERT → +ML) for the HCC and Multi test sets. The silhouette coefficient and Davies–Bouldin (DB) index are reported on the standardized 422-dimensional feature space (deterministic) and on 2-D t-SNE embeddings (mean ± SD over multiple random seeds, addressing t-SNE seed sensitivity). Across stages the silhouette increases monotonically and the DB index generally decreases, indicating a modest but consistent improvement in class structure; however, the absolute silhouette values remain low (the Kla and non-Kla classes substantially overlap), so the t-SNE is presented as qualitative support only rather than evidence of clear class separation.

**Dataset**	**Stage**	**Silhouette (422-D)**	**Davies–Bouldin (422-D)**	**Silhouette (2-D t-SNE)**	**Davies–Bouldin (2-D t-SNE)**
HCC	Sequence-only	0.0019	21.75	0.008 ± 0.002	22.38 ± 12.09
	+ProteinBERT	0.0030	17.69	0.011 ± 0.002	14.58 ± 2.57
	+ML	0.0071	11.53	0.066 ± 0.019	4.36 ± 1.36
Multi	Sequence-only	0.0029	19.39	0.005 ± 0.006	19.27 ± 11.89
	+ProteinBERT	0.0041	16.05	0.002 ± 0.0004	29.56 ± 4.64
	+ML	0.0089	10.62	0.020 ± 0.006	8.98 ± 3.95

Table S10

Table S10. Processed structural features of all 26 lysine residues in FAM210A (AlphaFold2 model AF-Q96ND0-F1; AlphaFold DB model version v6). pLDDT was extracted from the B-factor column (BioPython 1.87); SASA was computed with FreeSASA 2.2.1 (Lee–Richards algorithm, probe radius 1.4 Å) and normalized to the Gly-X-Gly maximum for lysine (230.0 Å²; Tien et al.) to obtain relative SASA (rSASA); secondary structure was assigned with biotite 1.6.0 (annotate_sse). Majority vote: Kla if ≥3 of 4 models predict lactylation; non-Kla if 0 models; borderline otherwise. The analysis script (analyze_fam210a.py) is available in the public repository.

**Site**	**pLDDT**	**SASA (Å²)**	**rSASA**	**Secondary structure**	**Pos count**	**Majority vote**
K33	47.6	188.9	0.821	Sheet	0	non-Kla
K44	45.7	185.1	0.805	Sheet	1	borderline
K53	44.9	199.1	0.866	Sheet	0	non-Kla
K66	45.2	180.8	0.786	Coil	1	borderline

K84	43.5	172.3	0.749	Sheet	0	non-Kla
K87	31.2	207.9	0.904	Sheet	4	Kla
K108	34.7	198.0	0.861	Coil	4	Kla
K109	42.8	184.3	0.802	Coil	3	Kla
K118	59.1	175.0	0.761	Coil	4	Kla
K127	81.8	141.1	0.613	Helix	2	borderline
K128	79.4	84.6	0.368	Helix	2	borderline
K135	83.8	193.3	0.840	Helix	0	non-Kla
K158	83.9	136.8	0.595	Helix	0	non-Kla
K180	73.4	117.7	0.512	Helix	0	non-Kla
K195	87.1	121.9	0.530	Helix	1	borderline
K214	82.4	158.6	0.689	Helix	4	Kla
K229	50.9	153.2	0.666	Helix	2	borderline
K240	83.2	112.4	0.489	Helix	2	borderline
K246	87.5	100.5	0.437	Helix	4	Kla
K251	88.8	131.9	0.573	Helix	4	Kla
K257	89.4	96.5	0.420	Helix	4	Kla
K262	86.2	146.9	0.639	Helix	4	Kla
K264	81.2	146.1	0.635	Helix	4	Kla
K268	70.3	151.7	0.659	Helix	2	borderline
K269	71.8	172.7	0.751	Helix	3	Kla
K270	67.6	175.9	0.765	Helix	3	Kla
